# Supplementary material for: Are carbon emissions trading and green financial instruments synergistic? -Comprehensive quantitative research based on content analysis
Source: PLoS One. 2024 Mar 7;19(3):e0298601. doi: 10.1371/journal.pone.0298601 (PMC10919601; doi:10.1371/journal.pone.0298601)
Supplement: S1 Data — (ZIP) [file pone.0298601.s001.zip › Data/地级市Z_F工作报告.zip!/╣π╢1⁄2/╖≡╔╜╩╨/└┤╫╘╣1⁄2╓┌║┼í╛┬φ┐╦╩2╛▌═°í┐_2016.docx]

各位代表：

　　现在，我代表市人民政府向大会报告政府工作，请予审议，并请政协各位委员和其他列席人员提出意见。

“十二五”时期和2015年工作回顾

　　“十二五”时期是我市加快转型升级的关键时期。市政府在省委、省政府和市委的正确领导下，在市人大、市政协的监督支持下，主动适应经济发展新常态，积极应对各种压力挑战，全力稳增长、调结构、促改革、惠民生、防风险，较好地完成了“十二五”规划各项目标任务，全市经济社会发展再上新台阶。

　　——这五年，是综合实力显著增强的五年。2015年实现地区生产总值8003.92亿元、人均地区生产总值10.83万元，分别比2010年增长55%、47.7%，地区生产总值五年跨越3个千亿元大关，在2015年全国大中城市中排名第16位。实现规模以上工业总产值19774.93亿元、增加值4406.95亿元，分别比2010年增长76.6%、69.5%。实现全社会固定资产投资3035.52亿元、社会消费品零售总额2687.22亿元、外贸进出口总值657.2亿美元，分别　比2010年　增长76.5%、87.1%、27.2%，其中出口482.1亿美元，增长45.9%。实现财政总收入1474.8亿元、地方一般公共预算收入557.43亿元、每平方公里税收0.33亿元，分别比2010年增长41.8%、82.2%、57.1%。在2015年全国科学发展综合实力百强区中，佛山5个区均位列前50强[1]。

　　——这五年，是产业转型成效突出的五年。经济结构持续优化。三次产业比例由2010年的1.9:62.6:35.5调整为2015年的1.7：60.5：37.8。工业发展提质增效。实施“两化”深度融合与金融科技产业融合发展战略，成为国家制造业转型升级综合改革试点。抢抓珠江西岸先进装备制造产业带龙头机遇，加快建设万亿规模先进装备制造业产业基地，引进一汽大众、北汽福田、中国中车等一批龙头项目。2015年先进制造业、高技术制造业占规模以上工业增加值比重分别为35.6%、7.3%，比2010年提高5.4、2.2个百分点。第三产业发展提速。2015年第三产业增加值增长10.3%，增速超过第二产业2.7个百分点。145个重大服务业项目完成投资1576.2亿元，广东工业设计城、佛山创意产业园等14个园区入选省级现代服务业集聚区，西樵山、长鹿旅游休博园获评国家5A 旅游景区，岭南天地成为广东岭南文化旅游休闲胜地，顺德区荣获联合国“世界美食之都”称号。农业现代化进程加快，成为全国首批农业产业化示范基地。创建国家创新型城市。2015年全社会研发经费支出占地区生产总值比例预计高于2.6%，比2010年提高0.95个百分点。五年专利申请量和授权量分别达13.97万件、10.3万件，均比“十一五”时期增长超65%，累计有效注册商标总量翻番。实施人才强市战略，2015年高技能人才达16.8万人，比2010年翻两番。全市共有博士后工作站59家，居全国地级市之首。推进金融创新“九个一”工程　[2]，2015年全市共有上市公司43家、股权投资基金（创投公司）276家、小额贷款公司39家，分别比2010年增加16家、220家和31家。重大产业平台扩容提质。佛山国家高新区形成汽车及零部件、高端装备制造、新材料等产业集群，产业集聚引领效应突出。中德工业服务区成为省六大重大发展平台和对德（欧）合作重要载体。广东金融高新区累计吸引252个金融企业和项目入驻，总投资额达525亿元，“金融后援基地+产业金融中心”态势显现。民营经济稳步发展。2015年民营工业对全市工业增长贡献率达81.8%，比2010年提高20.8个百分点。完成节能减排任务，2015年单位地区生产总值能耗及化学需氧量、二氧化硫、氨氮和氮氧化物排放量均比2010年下降20%以上。

　　——这五年，是城市品质稳步提升的五年。实施“产城人”融合发展战略，成为全国文明城市和国家信息惠民试点城市。完成城市升级三年行动计划，共投入1709.79亿元，建成项目316个，城乡面貌焕然一新。“强中心”战略成效显著，组团城市建设提速，岭南魅力小镇串珠成线。中德工业服务区成为全国首批“中欧城镇化合作示范区”，市图书馆、科技馆、青少年宫等文化新地标建成使用。交通体系日益完善，“两环五横四纵”[3]高速公路网基本建成。2015年中心城区公交分担率达35.5%，比2010年提高23.5个百分点。“三旧”改造五年新增改造项目925个，用地面积7.07万亩，已完成项目367个，用地面积1.84万亩。成为全国绿化模范城市，市域森林覆盖率达34.8%。实行最严格环境保护和治理机制，在全省率先成立“环保警察”。2015年全市　PM2.5、PM10平均浓度分别比2013年下降26.4%、30.1%[4]，22条重点整治河涌水质达V类标准。

　　——这五年，是改革开放深入推进的五年。完善大部门制和简政强镇改革，优化市、区、镇（街）事权财权配置，基层发展活力增强；市、区两级取消和调整行政审批事项995项，占全部审批事项的48%；建成四级行政服务中心体系和网上办事大厅，推进行政审批标准化，行政效能大幅提高；实施商事制度、投资管理体制和公共资源交易管理改革，强化社会信用体系和市场监管体系建设，成为省法治化国际化营商环境试点城市。财政预算管理和绩效管理进一步完善。率先完成事业单位分类改革试点工作，公立医院分类改革有序推进。国有企业改革重组有新进展。农村综合改革进一步深化，“两分离”改革、“两平台”建设成效明显，“两证”发放[5]基本完成。在全省率先构建公共法律服务体系，基层社会治理法治化成效初显。开放合作取得新进步，五年累计实际利用外资120.58亿美元、境外中方直接投资11.7亿美元，分别比“十一五”时期增长44.3%、711.9%。广佛同城化成果丰硕，广佛肇城际轨道佛肇段、广明高速西樵至陈村段等建成通车，广佛肇（怀集）经济合作区发展总体规划上升为省级发展战略。成为国家首批落实CEPA示范城市。积极对口帮扶云浮、清远，援疆援藏、扶贫开发“双到”工作成效突出。

　　——这五年，是社会民生不断改善的五年。全市财政民生支出五年累计达1654亿元，比“十一五”时期翻番，占一般公共预算支出的62.7%。每年省、市民生实事圆满完成。2015年城镇、农村常住居民人均可支配收入分别达3.98万元、2.21万元，比2010年增长67.3%、77.1%。五年新增就业45万人。建立健全城乡居民基本养老、生育、大病保险和疾病应急救助制度。企业退休职工基本养老金每年递增10%以上，近50万人受惠。实现省内异地就医即时结算、企业职工跨区享受居民门诊医保。五年建成保障性住房3.26万套，已安置2.85万套。成为全省首个教育综合改革试点市、推进教育现代化先进市。新市民随迁子女入读公办学校比例保持在70%以上，惠及近41万名学生。建立健全基本医疗卫生制度，2015年人均公共卫生服务经费比2010年增长60%，社区卫生服务中心实现镇（街）全覆盖。五年新建社区体育公园144个，农家书屋实现行政村全覆盖。平安佛山建设深入推进，2015年“110”日均刑事警情比2010年下降45.7%，5个镇（街）获评“全国安全社区”，里水镇成为“国际安全社区”。生产安全事故起数、事故死亡人数比“十一五”时期分别下降17.4%、12.1%。完成食品（农产品）安全示范市建设重点任务，升级改造农贸市场268个，建成市级以上“菜篮子”基地45个、食品安全示范点1285家，在全省率先开展肉品统一冷链配送和家禽集中屠宰试点。连续第7次荣获“全国双拥模范城”称号。

　　——这五年，是政府建设全面加强的五年。实施建设人民满意政府“1+11”行动计划，群众满意度不断提升。市、区两级政府部门权责清单在全省率先公布实施。健全科学民主依法决策机制，出台重大行政决策征求公众意见、专家咨询论证办法，成为拥有地方立法权的城市。根据2014年中国政法大学发布的中国法治政府评估报告，我市法治政府建设排名全国第3。主动接受人大依法监督、政协民主监督、司法监督和社会监督，办理人大代表建议428件、政协提案1034件，办复率达100%，市政府领导牵头办理议案、建议成为常态。贯彻落实中央“八项规定”、国务院“约法三章”等规章制度，深入开展党的群众路线教育实践活动和“三严三实”专题教育，切实整治“四风”问题，干部工作作风不断改进。

　　老年人、残疾人、青少年、妇女儿童事业，审计、粮食储备、应急管理、民族宗教、台湾事务、档案方志、社会科学、人防、气象等工作取得新成绩。

　　2015年是全面完成“十二五”规划的收官之年。市政府按照中央、省和市委的总体要求与部署，努力实现经济社会平稳较快发展，为“十二五”划上圆满句号。

　　稳定经济增长措施有力。出台稳增长工作方案，实现有效投资不断扩大、进出口保持稳定、社会消费持续增长。省、市重点项目分别完成投资459.52亿元、222.71亿元。贯彻落实各项外贸稳增长政策，鼓励企业参与“一带一路”建设，对沿线国家出口额810.46亿元，增长31.2%。推动外贸结构持续优化，一般贸易占出口总值比重达58.2%。推动连锁经营和社区商业发展，成为全国首批信息消费示范城市。商品住房去库存加快，销售均价下降6%，销售面积上升41.4%。服务企业务实深入。召开民营企业家大会，出台提振民营企业家信心促进创业创新40条措施。开展企业“暖春行动”，建立收集处理问题长效机制，切实解决企业发展困难。市、区两级共同设立企业融资专项资金15亿元。落实税收优惠政策，减免各项税收128.76亿元。下调堤围防护费和价格调节基金，减负5.2亿元。全市年主营业务收入超百亿元企业12家，其中超千亿元企业2家。

　　产业结构调整步伐加快。依托智能制造推动产业结构优化升级成为范例，受到国务院通报表扬[6]。出台《中国制造2025佛山行动方案》和《工业转型升级攻坚战三年行动实施方案》，开展智能制造发展专项行动，实施“百企智能制造提升工程”。中关村“互联网+智能制造”国际技术协同创新中心、密歇根大学国际智能制造创新中心等平台签约落户，广东省智能制造产业服务中心开业，机器人应用创新中心和产业创新联盟揭牌，全球工业机器人四大巨头[7]合作项目落户，全省首个机器人产业发展示范区在顺德区启动。装备制造业完成工业增加值1325.77亿元，增长15.5%。引进千山药机大健康产业基地、大族工业4.0高端装备工业园等一批先进装备制造项目，126家“工作母机”[8]制造业企业纳入省级骨干企业。完成工业技术改造投资386.15亿元，增长38.6%。全面实施“互联网+”行动计划，成功举办首届中国（广东）国际“互联网+”博览会、中国制造2025对话德国工业4.0大会。与中兴通讯签订大数据产业战略合作协议，建设大数据产业园等项目。现代服务业发展态势良好。毕马威大中华区后援中心、泛家居电商创意产业园等项目揭牌。成为国家物流标准化试点城市，国通保税物流中心（B型）通过国家验收。顺德区获评全省首个电子商务创新区。旅游文化创意产业发展提速，南庄生态休闲工程、美的鹭湖森林度假区、亚拉巴海创意生态农博城等60个重点项目建设加快，总投资额982.33亿元。

　　创新驱动发展战略深入实施。佛山国家高新区获批建设珠三角国家自主创新示范区，荣获中国产学研合作创新与促进奖。互联网+创新创业示范市建设启动。完善自主创新政策体系，出台贯彻落实省科技创新政策意见的16条措施。实施高新技术企业培育和企业研发机构建设专项行动方案，新增国家高新技术企业98家，总数达716家。产学研合作成果丰硕。全市共有省级新型研发机构25家，华南智能机器人创新研究院、佛山智能装备技术研究院挂牌成立。顺德区与国家工信部信息中心签约共建国家级产业众创空间，打造全国首个创客大学。全市新增省级、市级创新团队18个。省市共建研究生联合培养基地，推动佛山科学技术学院打造广东省高水平理工科大学。启动建设广东“互联网+”众创金融示范区，联合深圳创新投资集团设立百亿元规模的创新创业产业引导基金。广东金融高新区股权交易中心注册登记企业达1614家，帮助企业融资326亿元。在全省率先出台全面性债券融资扶持政策，“政银保”合作农业贷款模式全面推广。

　　城市环境得到优化提升。启动城市升级两年延伸行动计划。禅城奇槎片区、绿岛湖都市产业区建设加快。南海金融公园建设收尾，千灯湖公园获2015年全球城市开敞空间奖第一名。中德工业服务区坊塔主体结构封顶，广东（潭洲）国际会展中心、市妇女儿童医院奠基动工。顺德新城基础设施建设加快，区域核心带动作用增强。高明西江新城获“2015创建生态文明标杆城市”称号。三水北江新区加速成型，三水新城初具雏形。实施百村升级行动计划，完成203项古村落活化、241项城中村（旧社区）升级改造和508项“五好”新农村[9]项目建设。逢简村获2015中国最美村镇“榜样奖”。南海区和狮山镇成为第二批国家新型城镇化综合试点。加快城市基础设施建设。江罗高速佛山段、广明高速西延线完工，魁奇路西延线、乐龙公路一期等19条“断头路”建成通车。佛山西站、佛山地铁二号线一期、南海新型交通系统建设顺利。光纤入户率达52%，新建公共场所AP接入点1.5万个。与华为签订战略合作协议，推进“智慧佛山”建设。公交专用道总里程达102.6公里，新增公交车735辆。启动海绵城市建设，清淤排水管1869公里。基本完成100项环保民生实事。淘汰黄标车5.5万辆，完成电力行业“超洁净排放”改造工程，整治淘汰小锅炉495台，治理VOCs[10]排放重点企业95家。基本完成42条“一河一策”河涌整治和104个村级工业区环境整治试点，广佛交界区域水环境整治成效在省“四河整治”[11]第三方评估中位居前列。启动“绿城飞花”主题绿化景观建设，新建和改造半月岛湿地公园、桂畔湖湿地公园等花色主题景观项目11个。

　　改革开放持续深化。出台复制推广自由贸易试验区改革创新试点经验实施意见，在全国率先全面实施“互联网+易通关”。“一门式、一网式”政府服务改革全面推行，入选“2015全国社会治理创新最佳案例”。实现工商登记“三证合一、两证一章同发”[12]。完善网上办事大厅建设，市、区两级网上服务事项5938项，达到一级、二级、三级办事深度分别占95.6%、92.9%、60.4%[13]。粤港澳合作高端服务示范区成为“粤港澳服务贸易自由化示范基地”。成功举办粤桂黔高铁经济带合作试验区（广东园）建设现场会。广佛同城迈入新阶段。南海、顺德、三水区分别与广州市荔湾、番禺、花都区签订协议，共建合作示范区。海华大桥动工建设，广州地铁七号线西延至顺德区调整方案获国家发改委批准。与中山、江门签署合作协议推动佛中江协同发展。高明区与肇庆高要、江门鹤山、云浮新兴联手打造“百里西江旅游文化圈”。强化佛山云浮一体化帮扶，佛山（云浮）产业转移工业园累计入驻项目265个，总投资额404.14亿元。联合德国不莱梅、汉诺威等国内外城市组建“中德工业城市联盟”。

　　人民满意政府建设扎实推进。完成人民满意政府建设行动计划204个年度重点项目。根据第三方调查评估，2015年建设人民满意政府总体评价83.81分，比2014年提高1.35分。社会民生事业加快发展。建成城市社区公共服务“15分钟服务圈”。在全省率先实现城乡统一低保补差水平，失业保险城乡无差异化，大病保险参保人整体待遇提高17%。成为国家特殊教育改革实验区、国家现代学徒制试点市、全省首个所有区（县）通过国家“义务教育发展基本均衡区”认定的城市，现代职业教育体系建设改革试点获联合国“2015中国可持续发展城市范例”奖。顺利通过国家卫生城市复审，实现医师多点执业备案制管理，非公立医院占医院总数比例近半。启动创建国家公共文化服务体系服务示范区，南海区成为“中国曲艺之乡”，丹灶镇获评全国文明镇。成功承办2015广东国际旅游文化节，秋色欢乐节、“佛山韵律·和风鸣畅”等公共文化活动精彩纷呈。成为国家体育产业联系点城市，成功申办2019年国际篮联男篮世界杯。实施新市民积分制，2.69万名外来人员通过积分入户、人才引进等方式落户佛山。平安佛山建设成效明显。治安立体化防控体系逐步完善，“3+2”专项打击行动[14]位居全省前列，社会治安形势持续向好。实施安全生产“三大行动计划”[15]，创建安全生产标准化企业5663家，生产安全事故起数下降22.9%。创建省食品安全城市试点，设立“食品药品警察”。登革热疫情防控效果显著，积极应对龙卷风等自然灾害。根据北京师范大学发布的2015中国民生发展指数报告，佛山排名全国地级市第5。

　　各位代表！我市取得的每一项成绩，都是在省委、省政府和市委正确领导下，在市人大、市政协监督支持下，全市人民共同奋斗的结果。在此，我代表市政府向全市广大干部群众，中央、省驻佛山单位，驻佛山人民解放军指战员，武警官兵和社会各界人士表示崇高的敬意！向长期关心支持佛山发展建设的港澳台同胞、海外侨胞、国内外友人表示衷心的感谢！

　　在肯定成绩的同时，我们清醒地认识到，我市经济社会发展仍然面临不少问题和挑战，突出表现在：经济发展面临较大下行压力，产业发展层次总体不高，企业生产经营压力增大，转变发展方式任务依然艰巨；创新驱动发展基础仍不牢固，企业自主创新能力有待增强，社会创新创业活力还需进一步激发；城市建设水平与先进城市尚有差距，城市精细化管理能力亟待提升；　资源环境约束问题突出，大气和水环境综合治理任务较重，生态文明建设有待加强；教育、医疗、交通等民生社会事业还存在薄弱环节等等。这些问题和挑战需要我们认真研究，切实加以解决。

“十三五”时期奋斗目标和主要任务

　　“十三五”时期是佛山加快转型升级、深化改革开放的战略机遇期。从国际看，世界经济仍处于深度调整之中，但新一轮科技革命和产业变革正在创造历史性机遇。从国内看，尽管我国经济运行整体压力较大，但经济发展长期向好的基本面没有变，经济韧性好、潜力足、回旋余地大的基本特征没有变，经济持续增长的良好支撑基础和条件没有变，经济结构调整优化的前进态势没有变。从佛山看，我市发展虽然存在诸多问题和挑战，但也面临“互联网+智能制造”、新型城镇化、广佛同城化、粤桂黔高铁经济带、“一带一路”等机遇。面对新形势，我们要坚定发展信心、保持战略定力，科学谋划经济社会各项工作，走出一条质量更高、效益更好、结构更优的新路，对全省经济增长和结构调整起支撑作用。

　　“十三五”时期政府工作的指导思想：深入贯彻落实党的十八大和十八届三中、四中、五中全会精神，牢固树立创新、协调、绿色、开放、共享发展理念，按照中央、省和市委的决策部署，以提高发展质量和效益为中心，以全面深化改革为内在动力，以创新驱动发展为核心战略，以开放合作为关键路径，加快供给侧结构性改革，提升城市价值品质，加强生态文明建设，促进社会民生事业发展，确保全面建成高水平小康社会。

　　“十三五”时期经济社会发展的主要奋斗目标：全市地区生产总值年均增长7.5%，2020年达1.15万亿元；人均地区生产总值年均增长7%；　全社会研发经费支出占地区生产总值比重达2.9%；　居民人均可支配收入年均增长7.5%；城镇登记失业率控制在3%以内；单位地区生产总值能耗下降幅度完成省下达任务。

　　为此，必须着力做好以下五个“突出”：

　　一是突出创新引领发展。大力实施创新驱动发展战略，创建国家创新型城市，加快形成以市场为导向、以科技创新为核心、以企业为主体的开放型区域创新体系。发挥佛山改革先行优势，坚持以问题为导向，在重点领域和关键环节大胆推进改革创新，走佛山特色的改革发展之路。

　　二是突出统筹协调发展。以产业协调发展提升经济质量效益，推动传统优势产业与战略性新兴产业，先进制造业与现代服务业，国有、民营与外资等多种所有制经济协调发展，加快构建现代产业体系。以区域协调发展、物质文明和精神文明协调发展提升城市综合竞争力，建设宜居宜业的现代化大城市。

　　三是突出开放合作发展。建设高水平开放型经济体系，运用国际国内两个市场、两种资源，促进要素有序流动、资源高效配置、市场深度融合。参与“一带一路”建设，推进广佛同城化及粤桂黔、佛港澳等区域合作，增强对粤西地区辐射带动作用，努力把区位优势转化为区位责任，争创开放合作新优势。

　　四是突出绿色永续发展。树立生态文明理念，以提高环境质量为核心，构建政府、企业、社会共治的环境治理体系，创造优良人居环境。全面节约和高效利用资源，建立绿色低碳生产生活体系，建设国家生态文明示范城市和国家森林城市，实现与全面建成高水平小康社会相适应的环境质量目标。

　　五是突出共建共享发展。把保障和改善民生作为经济社会发展的出发点和落脚点，着力提高居民收入，完善社会保障，逐步扩大多层次、广覆盖的基本公共服务供给，促进民生事业繁荣发展。营造机会公平的社会环境，保障平等参与、平等发展权利，使市民群众拥有更多幸福感和获得感。

2016年工作安排

　　2016年是“十三五”开局之年，也是推进供给侧结构性改革的攻坚之年。我们要按照市委十一届七次全会的总体部署，坚持稳中求进、稳中提质，着力保持经济中高速增长与社会和谐稳定，为实现“十三五”规划开好局、起好步。

　　今年经济社会发展的主要预期目标是：全市地区生产总值增长8.0%-8.5%，人均地区生产总值增长7.0-7.5%；地方一般公共预算收入增长9.5%；　全社会固定资产投资增长13%；社会消费品零售总额增长10%；出口总额增长1.5%；居民人均可支配收入增长与经济增长基本同步；城镇登记失业率控制在3.5%以内；　居民消费价格涨幅控制在3.0%左右；　单位地区生产总值能耗、主要污染物排放量下降幅度完成省下达任务。

　　实现上述目标，重点做好以下八方面工作：

　　一、以智能制造为主攻方向，加快产业转型升级

　　加快供给侧结构性改革，构建先进制造业和现代服务业双轮驱动的现代产业体系，增强经济发展新动力。

　　大力发展“互联网+智能制造”。建设中国“互联网+智能制造”试点城市，走好“工业2.0”补课、“3.0”普及和“4.0”示范[16]的转型升级之路。重点发展智能数控装备、工业机器人、智能家电等智能装备和产品，培育一批“中国制造2025”试点示范企业，打造柔性化生产、场景化应用的示范工厂。建设智能制造产业园，打造具有核心竞争力的机器人产业。探索制定区域智能产品技术标准，推动佛山智能产品兼容互通，打造“佛山智造”品牌。全面落实“互联网+”行动计划，推动互联网新技术、新理念、新模式与制造业跨界融合，发挥“中国在线制造”云服务平台带动作用，发展基于工业互联网的众包设计、云制造等新型制造模式，促进制造业向智能化、绿色化、高端化发展。加快云计算、大数据、物联网等产业发展，推动浪潮集团云计算中心、中兴通讯大数据产业园及华南大数据研发平台等项目建设。办好第二届中国（广东）国际“互联网+”博览会。

　　加快发展先进装备制造业。以承办第二届珠江西岸先进装备制造业投资贸易洽谈会为契机，加快建设珠江西岸先进装备制造业产业创新基地。以“工作母机”型装备制造业为重点，提升发展陶瓷机械、木工机械、塑料机械等优势行业，培育引进一批创新能力强、经济效益好的重点项目，打造装备制造业全产业链。积极参与构建装备制造标准体系，鼓励企业加强协同合作，培育一批装备制造产业配套优势企业。推动一汽大众二期、千山药机大健康产业基地等项目建成投产。以省战略性新兴产业基地为平台，打造高端新型电子信息、半导体照明、生物医药等产业集群，推动战略性新兴产业集聚发展。

　　优化提升传统优势产业。支持传统优势产业开展智能化成套装备和自动化生产线改造，加快机器人推广应用。落实省工业技改事后奖补、首台（套）重大技术装备及先进装备保险补偿机制等扶持政策，完成工业技改投资增长35%以上。加快专业镇转型升级步伐，推动陶瓷、家电、金属加工、家具等优势产业链协同延伸，发展区域性产业联盟，创建国家级知名品牌示范区，打造“佛山家居”品牌。完善现代农业生产服务体系，大力发展高附加值、集约化生产的互联农业、品质农业和生态农业。

　　提高现代服务业发展水平。大力发展产业金融、现代物流、电子商务等生产性服务业，加快省级现代服务业集聚区和重大服务业项目建设，争创国家服务贸易特色出口基地，推动生产性服务业向专业化和高端化延伸。推进制造业服务化，鼓励企业围绕产品功能拓展专业增值服务，开展全生命周期管理[17]等模式创新，促进生产型制造向服务型制造转变。依托广东工业设计城、顺德创意产业园等载体，做强工业设计产业，建设“工业设计之都”。以建设广东（潭洲）国际会展中心、佛山西站会展中心为契机，发展壮大会展产业链，塑造会展品牌。大力发展旅游文化、休闲娱乐、健康养老等生活性服务业，促进生活性服务业向精细化和品质化转变。鼓励线上线下互动，推动实体商业创新转型。建设西樵山国艺影视城、华侨城文化旅游综合体等重点项目，支持石湾西片区创建国家级文化产业示范园区，促进旅游文化创意产业融合发展。

　　着力促进“三去一降一补”。围绕去产能、去库存、去杠杆、降成本、补短板，制定实施“去、降、补”行动方案，打好供给侧结构性改革攻坚战。把处置“僵尸企业”作为化解产能过剩的“牛鼻子”，建立规范有序的企业退出机制，推动低效企业兼并重组、债务重组、破产清算，促进优胜劣汰和市场出清。支持居民自住和改善型住房需求，落实稳定住房消费政策，完善公积金异地贷款机制，引导发展都市型产业地产、分享型众创空间、综合性养老社区等业态，政企联动、多措并举化解房地产库存，促进房地产市场健康稳定发展。落实中央和省去杠杆政策措施，做好金融风险防范工作，营造良好金融生态环境。落实各项税费优惠减免政策，切实降低制度性交易成本、税负成本、企业运营成本和财务成本等，2016年为全市企业减负约280亿元[18]。加快提升制造业发展水平和服务业规模层次，增强企业自主创新能力，补齐经济发展短板。提高供给体系质量和效率，实施创建全国质量强市示范城市工作方案，扎实开展质量标杆和领先企业示范活动，推广先进质量技术、工艺和管理方式，培育一批具有国际竞争力的品牌企业和名牌产品，增加中高端产品和优质服务有效供给。

　　助推民营经济提质增效。落实提振民营企业家信心促进创业创新40条措施，常态化开展各类暖企行动，帮助企业破解用工、用地等发展难题。引导民营企业建立现代企业制度，完善法人治理结构，学习先进管理方法，强化对“创二代”的培训。鼓励民营企业通过参与混合所有制改革、兼并重组、向产业价值链两端延伸等方式做强做优，发展壮大一批百亿规模龙头企业，促进小企业提升规模层次，营造“大企业顶天立地，中小企业铺天盖地”的产业生态。

　　二、推动大众创业万众创新，汇聚科学发展新动能

　　以创新驱动发展战略作为经济工作总抓手，促进金融科技产业深度融合，努力形成以创新为引领的科学发展新格局。

　　加快创建国家创新型城市。积极参与珠三角国家自主创新示范区和全面创新改革试验试点省建设。依托佛山国家高新区、广东金融高新区、中德工业服务区、禅西产业轴、南方智谷等高端平台，打造佛山特色区域创新走廊。加快构建以企业为主体、市场为导向、产学研用相结合的自主创新体系。落实各项普惠性科技政策，完善科技创新投入机制，提高财政资金使用效益，带动社会资本参与创新创业。注重开发引领产业变革的颠覆性技术，强化科技创新对产业中高端发展的支撑作用。健全科技成果转化和技术转移机制，加速创新成果市场化、产业化。

　　强化企业创新主体地位。大力培育高新技术企业，落实税收优惠政策和扶持措施，力争2016年全市高新技术企业达1000家。实施规模以上高新技术企业和创新型企业研发机构全覆盖行动，推动规模以上工业企业组建工程中心、重点实验室，支持骨干企业创建国家级企业研发机构和海外研发机构，力争2016年规模以上高新技术企业、工业企业建有研发机构率分别达80%、20%。加快建设佛山中国科学院产业技术研究院等新型研发机构，引进一批高水平研发平台，推进产业关键共性技术攻关。发展重点行业产业技术创新联盟，建设区域性、行业性研发中心、质检中心和信息中心。加快专业镇转型升级，完善中小微企业公共服务体系，建设一批产学研协同创新中心和创新型产业集群，推进专业镇跨区域创新合作。

　　强化金融服务创新。建设广东“互联网+”众创金融示范区，构建“互联网+金融”服务体系，打造众创、众包、众扶、众筹平台。发挥广东金融高新区股权交易中心、全景网（广东）路演中心等平台作用，推动佛山民间金融街升级为众创金融街。完善中小微企业投融资机制，发挥15亿元企业融资专项资金作用，研究设立政策性担保公司和融资担保基金，缓解中小微企业融资困难。用好100亿元创新创业产业引导基金，支持互联网企业创新创业和传统产业升级改造。鼓励企业利用多层次资本市场进行资本运营，提高直接融资比重。

　　优化创新创业环境。以建设互联网+创新创业示范市为契机，以佛山互联网+创新创业产业园为重要载体，强化创新服务改革，打破政策、行业和技术壁垒，推动各类创新要素开放集聚共享，把个体式创业、孤岛式创新汇聚成大众创业、万众创新的洪流。实施科技企业孵化器倍增计划，完善“创业苗圃—孵化器—加速器—专业园区”四级孵化育成体系，高标准建设佛山国家火炬创新创业园、瀚天科技城、新媒体产业园等国家级孵化器。打造一批国内一流的专业化、集成化、开放式创客空间和创业服务平台，营造低成本、便利化、全要素的综合创业服务环境。支持分享经济发展，鼓励大中型企业通过生产协作、开放平台、共享资源、开放标准等方式，带动上下游小微企业和创业者共同发展。弘扬创新文化，营造敢为人先、宽容失败的良好氛围，激发企业家创业创新精神。

　　夯实创新发展人才基础。实施高层次创新人才引进计划，着重引进高水平创新科研团队和行业技术人才。实施科技型企业家成长计划，引导本土企业家开展技术变革、管理变革和二次创业，培育一批具有国际化视野和现代管理理念的职业经理人。实施技能人才培养计划，弘扬精于设计、工于制造的“工匠精神”，培育一批现代产业工人。推动佛山科学技术学院创建广东省高水平理工科大学，加快新校区建设，构建“大学+龙头企业+一流科研院所机构”新模式。扎实创建现代职业教育综合改革示范市，加快研究生联合培养基地、北京外国语大学南方（佛山）国际学院、中德工程学院等建设，完善从中职、高职、应用本科到专业学位研究生的人才培养模式。

　　三、全面深化改革，营造法治化国际化便利化营商环境

　　坚持向改革要红利，突破体制机制束缚，大力推进国家制造业转型升级综合改革试点，培育可持续发展新优势。

　　打造不是自贸区的“自贸区”。主动对接广东自贸区建设，复制推广自贸区改革经验和先进做法，构建与国际标准对接的投资便利规则体系、贸易便利化体系和金融改革创新体系，努力成为自贸区的延伸区、联动区。全面推进“互联网+易通关”改革，降低企业通关门槛和成本。积极探索开展泛家居市场采购新型贸易方式。发挥国通保税物流中心（B型）服务优势，完善跨境电商综合配套服务，促进跨境电商产业发展。

　　进一步转变政府职能。全面推行权责清单制度，推进行政审批标准化建设。推动网上办事大厅与“一门式、一网式”政府服务改革深度融合，建设市民个人网页、企业专属网页，争创国家“互联网+政务服务”试点城市。深化商事制度改革，强化商事活动后续监管。健全企业投资“三单”管理和政府投资决策管理制度，完善政府基建投资项目代建模式。推进财政预算编制、公共资源交易监管机制和事业单位分类改革。完善社会信用体系建设。

　　创新社会治理体制。建立基层善治体系，完善镇（街）领导直联制度和村（居）民主议事制度，规范发展基层自治组织功能。完善信访工作制度，推动人民调解、行政调解、司法调解有机衔接，妥善化解社会矛盾纠纷。健全“三官一师”[19]联系村（居）制度，推进基层社会治理法治化。规范发展协会商会、社区服务等社会组织，加强社会工作专业人才队伍建设。

　　深化农村综合改革。加快农村土地承包经营权确权登记颁证，深化股权固化到户改革。规范农村集体经济管理，完善农村集体经济信息化监管网络，推广农村股权管理交易平台，建设覆盖区、镇（街）、村（居）的农村产权管理服务平台体系。探索农村住房抵押改革，认真做好南海区国家农村集体经营性建设用地入市改革试点工作。

　　四、强化区域合作共赢，构建高水平开放型经济体系

　　积极拓展区域发展新空间，努力在更宽领域、更深层次、更高水平上配置资源，再创对内对外开放新优势。

　　积极推动广佛同城化。实施《广佛同城化“十三五”发展规划》，构建基础设施共建共享、产业发展合作共赢、公共事务协作管理新格局。加强广佛轨道交通规划衔接，加快建设海华大桥、珠江大桥放射线接广佛新干线等项目，打通交界地区“断头路”。强化南海、顺德、三水区与荔湾、番禺、花都区深度合作，重点建设“五眼桥-滘口”和“三山-东沙”示范区。

　　加快区域一体化发展。以粤桂黔高铁经济带合作试验区（广东园）建设为依托，推动沿线城市在产业、环保、旅游等方面加强合作。把握泛珠三角经济合作和西江黄金水道扩能升级机遇，打造珠江-西江经济带江海联运重要枢纽。建设粤港澳合作高端服务示范区，拓展佛港澳合作新空间。落实中央和省下达的各项扶贫开发任务，开展对口帮扶云浮、湛江。

　　着力扩大对外开放。深化与“一带一路”沿线国家经贸、科技和文化合作交流，巩固发达国家市场份额，努力开拓新兴市场。引导企业布局“一带一路”沿线投资，完善扶持企业境外建设工厂、研发中心和营销网络的政策体系，鼓励有实力企业开展跨国投资。推动加工贸易转型升级，扩大高技术含量、高附加值产品出口。发挥中德工业城市联盟、中德对话论坛等平台作用，加快从德国及欧美等先进国家“引制、引智、引资”[20]步伐。

　　五、加强城市建设管理，推动城市功能品质提升

　　以高水平规划引领城市现代化，加快新型城镇化建设，完善城市治理体系，推动城市升级向城市升值转变。

　　加快城市升级步伐。继续推进城市升级两年延伸行动计划。实施“强中心”战略，加快城市中轴线和东平河水轴线建设，打造城市发展脊梁。促进城市组团协调发展，推动顺德北部片区一体化规划建设，加快禅城老城区改造以及听音湖片区、顺德新城、西江新城、北江新区等片区建设。加快省级新农村连片示范工程建设，建成一批宜居社区、特色古村落和美丽乡村，全面完成百村升级行动计划。完善“三旧”改造政策，引导“三旧”改造土地更多用于实体经济发展。支持南海区开展国家新型城镇化综合试点和省新一轮深化“三旧”改造综合试点。

　　加强基础设施建设。推动佛山地铁二号线一期、地铁三号线、广佛环线等项目建设，做好佛山地铁四号线、广州地铁七号线西延线、广佛江珠线等前期工作。加强地下空间开发利用。启动一环西拓工程，力争打通魁奇路东延线、汾江路南延线等20条“断头路”。加大能源、电网建设力度。推动光网城市建设，提高光纤网络、4G通信网络和公共场所WiFi覆盖率。

　　提高城市综合管理能力。实施大数据战略，推动公共数据资源开放共享，推进“三网融合”[21]和国家信息惠民、信息消费示范城市建设，建立全市统一的数字城管信息系统和智能交通指挥平台，建设“智慧佛山”。深入实施城市管理“四化”工程[22]和城市管理考评，加强城市精细化管理，全面提升城市整洁度、美观度。健全城市应急管理机制。实施公共交通优先发展战略，大力推进中心城区公交一体化，积极研究BRT[23]模式，优化公交资源和线网，实现公交提速及服务提升。

　　提升城市文化软实力。实施文化升级两年行动计划，弘扬陶瓷、武术、粤剧等岭南特色文化，加快公共文化设施建设，建设文化导向型城市。加强传统文化保护与利用，促进非物质文化遗产传承与发展。强化公共资源品牌保护。开展全民健身运动，推进2019年国际男篮世界杯场馆等体育设施建设，办好佛山国际龙舟赛等赛事。实施文明城市建设三年提升计划，深入开展文明村居、文明公交及“微文明”市民活动，打造乐善之城、志愿者之城。

　　六、推动绿色发展，打造美丽宜居城市

　　坚持绿色可持续发展理念，加强生态文明建设，筑牢生态安全屏障，构建人与自然和谐发展新格局。

　　狠抓环境综合治理。严格落实环境保护“一岗双责”责任制，建立健全评价考核和责任追究制度。开展“互联网+”环保建设，建立全市统一实时在线环境监控系统，提升监测监管执法能力。大力整治大气污染重点行业、机动车尾气污染及扬尘污染，实现空气质量持续改善。开展内河涌“一河一策”整治计划，抓好广佛跨界流域综合整治，努力实现建成区主干河涌基本消除黑臭。推进100个村级工业区环境整治提升。启动土壤污染防治，推进生活垃圾分类回收处理试点，加快工业危险废物处置设施建设。

　　发展低碳循环经济。实施能源和水资源消耗、建设用地等总量和强度双控行动，推动工业绿色发展，培育循环经济示范园区和示范企业，建设绿色社区示范点，发展节能环保产业。加快“3C”绿色电网[24]建设，推广电力需求侧管理城市综合试点经验，打造“互联网+”能源及智慧能源管理平台。推广应用节能环保技术和装配式建筑[25]，大力发展绿色建筑。倡导低碳出行，加快新能源汽车推广应用及配套充电设施建设。

　　加强生态体系建设。实施主体功能区规划，创建国家生态文明示范城市和国家森林城市。加强生态控制线、林业生态红线划定管理，重点生态功能区实行产业准入负面清单。开展海绵城市试点，推进地下综合管廊建设，实行低影响开发模式，增强城市水资源涵养及排水排涝能力。实施新一轮绿化佛山大行动，推进森林公园体系和“绿城飞花”主题绿化景观建设，建设“佛山一环”环城百里花廊等项目，建成一批特色主题公园、花色主题景观道路及滨河绿化景观带。

　　七、加大民生保障力度，促进社会事业发展

　　加快社会民生事业发展，多谋民生之利，多解民生之忧，推动群众幸福感与经济社会发展同步提升。

　　推进基本公共服务均等化。落实基层基本公共服务均等化实施方案。创建国家教育综合改革试验区，统筹优化城乡义务教育资源配置，推进各类教育协调优质发展。落实促进就业政策措施，做好高校毕业生、就业困难人员等群体就业服务，强化创业带动就业。开展医保城乡一体化改革，提高六类底线民生待遇水平[26]。完善社会福利和社会救助体系，发展各类养老服务机构和社区居家养老服务。推动卫生强市建设，深化医药卫生体制改革，探索公立医院综合改革，加快完善分级诊疗制度。创建国家公共文化服务体系示范区，完善城乡社区“10分钟文化圈”。以常住人口全覆盖、基本公共服务均等化为目标，建立健全为居住证持有人提供基本公共服务的工作机制。实施革命老区后续帮扶工程，夯实老区发展基础。

　　集中力量办好十件民生实事。一是提高城乡居民基本养老保险基础养老金和社会救助保障水平。二是全面完成省下达的保障性住房工作任务。三是提高市属高校生均综合定额拨款标准和公益普惠性幼儿园生均公用经费标准，扩大全市优质普通高中学校招收指标生范围。四是完成100项环保民生实事，新建污水管网300公里。五是加强基层医疗卫生机构综合服务能力建设。六是建设15项重点民生水利工程。七是中心城区公交分担率提升至40%。八是完成市工人文化宫新建项目，建设佛山全民健身中心、34个社区体育公园和150个行政村（社区）综合性文化服务中心。九是落实新市民随迁子女在我市参加中、高考政策，实行二代身份证免费办理居住证。十是新增公共场所AP接入点1.5万个，全市光纤接入用户达149万户，光纤入户率超65%。

　　全面推进平安佛山建设。健全立体化社会治安防控体系，严厉打击、严密防范各类违法犯罪活动，确保社会大局稳定。强化“党政同责、一岗双责、失职追责”责任体系建设，实施安全生产“五大行动计划”和村级工业区安全本质提升工程　[27]，推进安全监管模式改革创新，防范各类安全事故发生。推进省食品安全城市创建试点，实施“十大食品安全工程”[28]，建立健全最严格食品药品监管机制，保障市民“舌尖上的安全”。健全应急救援机制，预防和处置好各类突发公共事件。做好国防动员、双拥、优抚安置工作，巩固军政军民团结。

　　八、强化政府自身建设，提高人民群众满意度

　　抓好人民满意政府建设，提升政府依法履职能力，尽心竭力为人民服务、对人民负责、让人民满意。

　　强化依法行政。依法接受人大及其常委会的监督，自觉接受政协的民主监督。严格按照法定权限和程序履行政府职能，积极稳妥推进政府规章制定工作，创建珠三角法治政府示范区。认真落实重大行政决策法定程序，建立健全重大决策终身责任追究制度及责任倒查机制。加强新型智库建设，健全决策咨询论证制度，提高科学民主依法决策水平。深化行政执法体制改革，加大食品药品、安全生产、环境保护、城乡建设等重点领域执法力度，积极推行综合执法模式，加强执法队伍建设，充实基层执法力量。支持审计机关依法独立行使审计监督权。深入推进政务公开，推动政府新闻发布常态化、规范化。

　　积极主动作为。加强整治领导干部“为官不为”，落实“三个区分”[29]，鼓励支持各级领导干部勇于担当、主动作为，提振党员干部信心。建立健全政府部门权责清单制度，建立政府部门行政问责、行政过错责任追究制度。健全重点工作任务分解落实机制和绩效考评机制，加大检查督办力度，确保事事有回音、件件有着落。

　　狠抓政风建设。深入践行“三严三实”，巩固和拓展党的群众路线教育实践活动成果，切实做到求实务实抓落实、正风正己尽责任。认真落实党风廉政建设主体责任。推进廉洁试验区建设，全面推行重大项目建设廉洁风险同步预防，建设党政机关廉政风险科技防控平台，筑牢制度“篱笆”，防范权力“越线”。

　　各位代表！“十三五”宏伟蓝图已经绘就，新的发展理念引领我们开启新征程。让我们以党的十八大和十八届三中、四中、五中全会精神为指引，在省委、省政府和市委的坚强领导下，凝心聚力、奋发有为，为开创我市改革开放和现代化建设新局面，共同创造佛山更加幸福美好的明天而努力奋斗！
